# Parents Appreciate Streamlined Care From Occupational Therapists for Their Child's Simple Hand Fracture: A Qualitative Study

**DOI:** 10.1177/22925503251414393

**Published:** 2026-02-05

**Authors:** Emily L.M. Dalton, Thomas R. Cawthorn, Yoga Dhanapala, Frankie O.G. Fraulin, A. Robertson Harrop, Karen Hulin, Rebecca L. Hartley

**Affiliations:** 1 4257 Queen's University School of Medicine, Kingston, Ontario, Canada; 2 Section of Plastic Surgery, 2129 University of Calgary, Calgary, Alberta, Canada; 3 Section of Pediatric Surgery, 2129 University of Calgary, Calgary, Alberta, Canada; 4 Department of Rehabilitation, 9978 Alberta Children's Hospital, Calgary, Alberta, Canada

**Keywords:** pediatric hand fracture, occupational therapist, nonoperative management, parental satisfaction, ergothérapeute, fracture de la main en pédiatrie, gestion non opérationnelle, satisfaction des parents

## Abstract

**Introduction:** Most pediatric hand fractures are treated nonoperatively, yet the majority of these children are managed by plastic surgeons. Our institution developed a protocol, deemed the Simple Fracture Protocol (SFP), which streamlines pediatric hand fracture care by redirecting patients with simple fractures to occupational therapists for management. This qualitative study evaluated parental satisfaction with the SFP. **Methods:** A semistructured telephone interview was administered to parents of children with hand fractures treated under the SFP between March 1, 2024, and May 31, 2024. Interviews were audio recorded, anonymized, and transcribed. Interviews were completed until data saturation was reached. Inductive content analysis was performed and reviewed by the research team to confirm key themes. **Results:** Of the 78 families contacted, 40 parents participated in the study. Parents reported high satisfaction with the care provided; 100% felt their care expectations were met. Four key themes were identified (1) communicating clearly, (2) creating a calm and comfortable environment, (3) setting expectations, and (4) streamlining clinical processes. Most patients, 67.5%, recovered within the expected 6 weeks. Delays in healing were often due to early return to sports or nonadherence to recovery guidelines. **Conclusions:** The SFP has been well-received by parents. Adjustments in the care instructions provided to patients partaking in high contact sports may further enhance the patient/family experience. This model offers a valuable framework for other institutions seeking to optimize pediatric fracture care.

## Résumé

**Introduction :** La plupart des fractures de la main en pédiatrie sont soignées sans être opérées, mais la majorité est tout de même prise en charge par des plasticiens. L’établissement des auteurs a préparé un protocole appelé *Simple Fracture Protocol* (SFP ou protocole pour les fractures simples) qui, pour rationaliser les soins de la main en pédiatrie, réoriente les patients victimes de fractures simples vers des ergothérapeutes. La présente étude qualitative évaluait la satisfaction des patients à l’égard du SFP. **Méthodologie **: Les parents d’enfants qui avaient été victimes d’une fracture de la main et qui avaient été soignés conformément au SFP ont participé à des entrevues téléphoniques semi-structurées entre le 1^er^ mars et le 31 mai 2024. Les entrevues étaient enregistrées, anonymisées et transcrites. Elles se sont poursuivies jusqu’à la saturation des données. L’équipe de recherche a procédé à une analyse inductive de contenu, qu’elle a révisée pour confirmer les grands thèmes retenus. **Résultats :** Parmi les 78 familles contactées, 40 parents ont participé. Les parents se sont dits hautement satisfaits des soins reçus. En effet, 100% ont déclaré que leurs attentes envers les soins avaient été respectées. Quatre thèmes sont ressortis : 1) des communications claires, 2) la création d’un environnement calme et agréable, 3) l’établissement d’attentes et 4) la rationalisation des processus cliniques. La plupart des patients (67,5%) se sont rétablis dans les six semaines. Lorsque le rétablissement était retardé, ce retard était souvent attribuable à un retour précoce aux activités sportives ou au non-respect des directives. **Conclusions :** Les parents ont bien accueilli le SFP. Des adaptations aux directives de soins transmises aux patients dont l’enfant participait à des sports de contact pourraient améliorer encore davantage l’expérience du patient et de sa famille. Ce modèle représente un cadre précieux pour les autres établissements qui cherchent à optimiser les soins des fractures en pédiatrie.

## Introduction

Previously at our institution, pediatric patients with hand fractures were all evaluated by a plastic surgeon. Those with simple fractures would then be referred to the occupational hand therapists (OT) for splinting and active range of motion exercises. This patient flow was inefficient as pediatric hand fractures are common,^1^ most can be managed nonoperatively,^1^ and having patients see both the plastic surgeon and the OT increases wait times for other referred patients.

**Figure 1.**
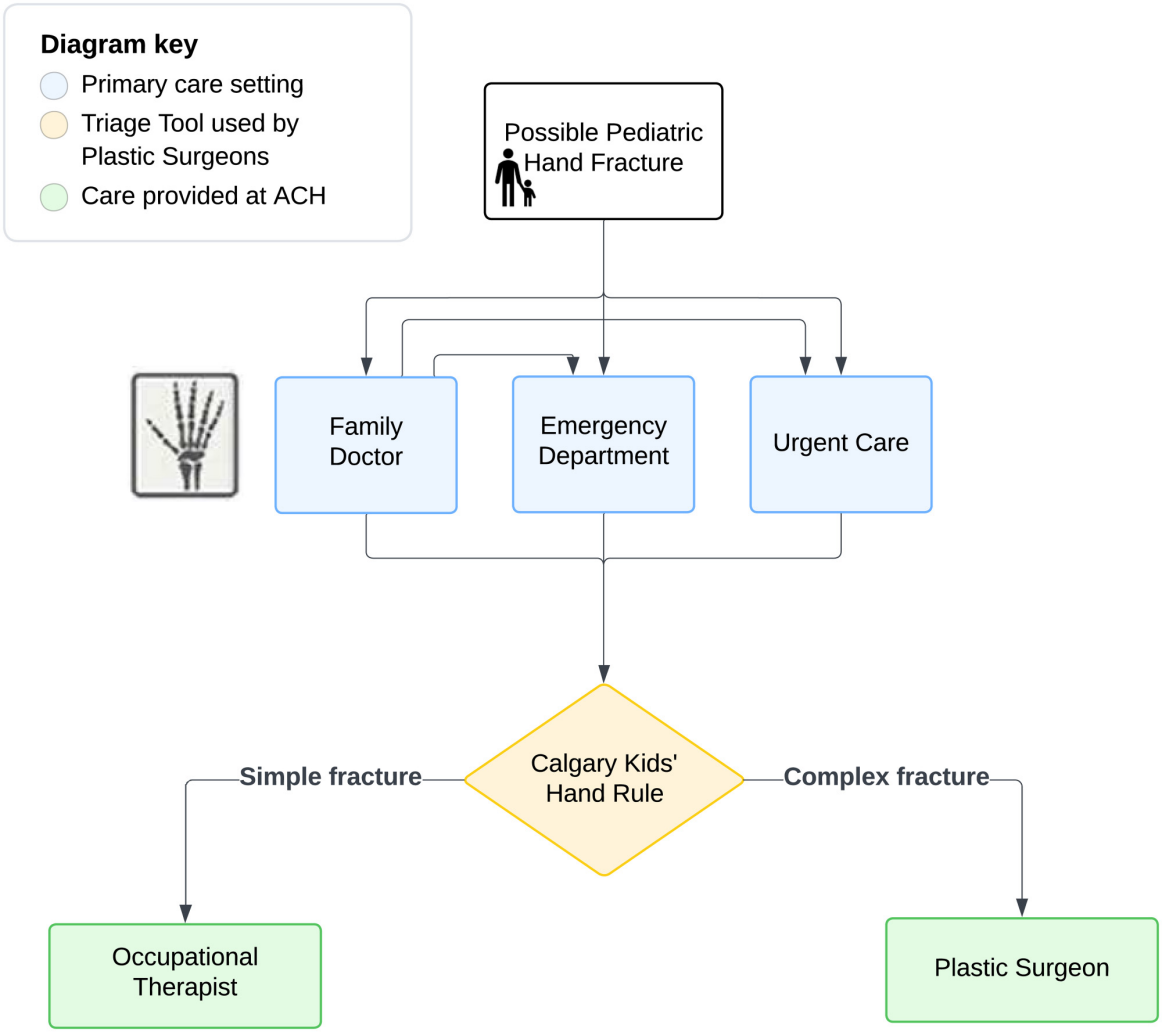
The Alberta Children's Hospital Simple Fracture Protocol care pathway.

**Figure 2.**
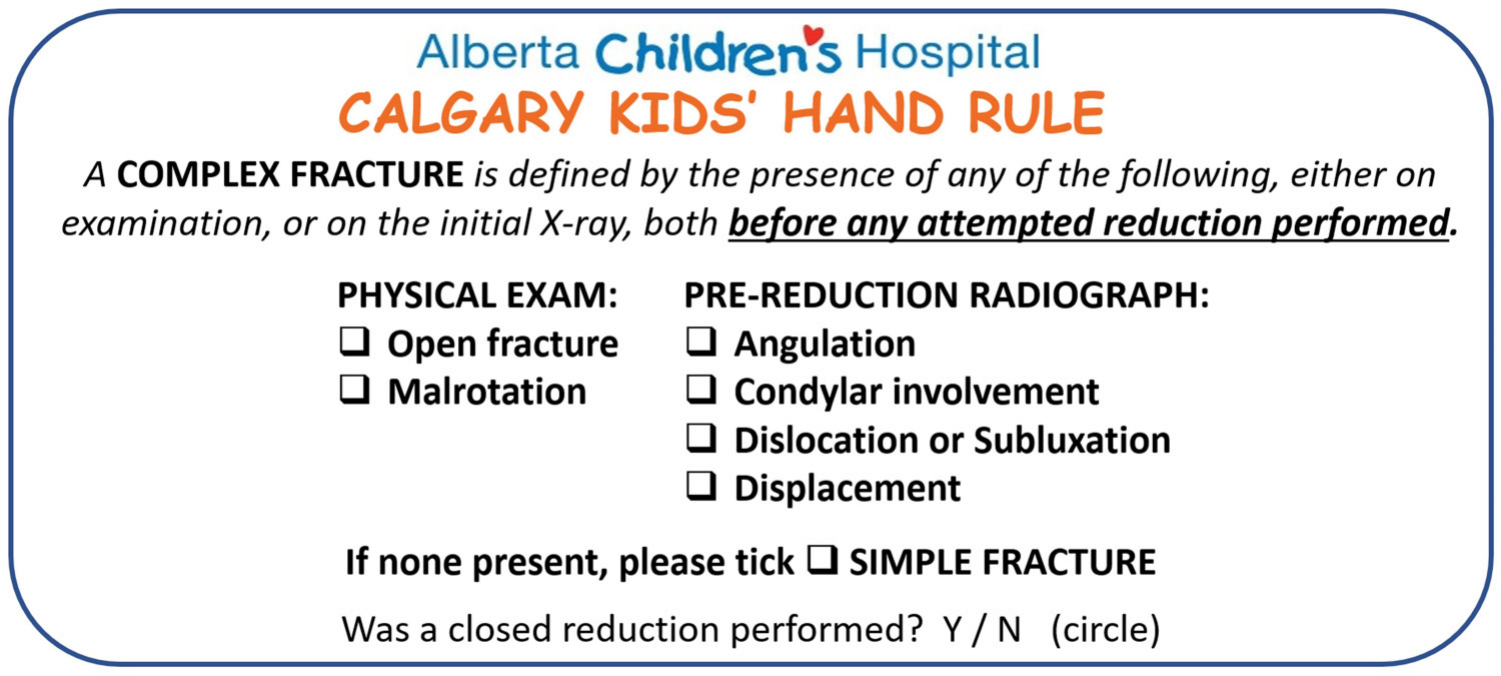
The Calgary Kids’ Hand Rule predictors.

To address this issue, our institution implemented a novel protocol for managing simple hand fractures, termed the Simple Fracture Protocol (SFP). The SFP appropriately matches provider expertise with patient needs (Figure 1). In this protocol, patient referrals are electronically triaged by the pediatric plastic surgeon into either simple or complex fractures based on the Calgary Kids Hand Rule (CKHR) (Figure 2).^2^ Using the CKHR, a simple fracture is defined as a closed fracture, without rotation, angulation <5 degrees, displacement <2 mm, and not associated with subluxation or dislocation.^2^ Essentially, a simple fracture is one where the fracture fragments remain in good anatomic position for healing. Fractures determined to be simple are redirected to the OT team to provide assessment and treatment, thus bypassing the plastic surgery consultation. The OT team is given carte blanche to reject any redirected fractures back to the plastic surgeon.

At their appointment (typically 45-60 min long), the OT makes a custom thermoplastic splint for the patient and then reviews the management plan with the patient and parent. This includes wearing the splint continuously for the first 4 weeks, only removing the splint to do active range of motion exercises 4 times per day. For weeks 5 and 6, the splint is only worn during activities where the patient could injure their hand. After 6 weeks, the patient should have full recovery, defined as pain free, full range of motion and able to return to all normal school and extracurricular activities without a splint. There is no follow-up appointment scheduled; instead, families are given the OT office contact information and encouraged to call with any questions or concerns.

The safety, effectiveness, and health care provider satisfaction of the CKHR has been previously evaluated^3^; however, parental satisfaction with the SFP—which is based on CKHR classifications—has not been studied. The purpose of this study was to explore parental satisfaction with the SFP. Understanding parental perceptions can identify areas for improvement in care delivery and help enhance the overall patient and family experience.

## Methods

### Design and Ethics

This qualitative study employed a semistructured interview designed by our research team to explore parent satisfaction with the SFP. This study was approved by our institution's Conjoint Health Research Ethics Board.

### Participant Selection and Setting

Purposive sampling was used to identify patients treated with the SFP between March 1, 2024, and May 31, 2024. Inclusion criteria: children with hand fractures referred to plastic surgery at our institution and triaged for management according to the SFP. Exclusion criteria: non-English-speaking patients. Between August 8, 2024, and November 8, 2024, the semistructured interview was administered via telephone to parents of children included in the study.

### Data Collection

Interviews were administered by a single research team member (ED). The interview was 5 to 10 minutes long, excluding the consenting process, and included both open and closed-ended questions asking parents to describe and elaborate on their experience with key aspects of the SFP (Appendix 1). Each phone interview was audio recorded, anonymized, and transcribed verbatim prior to being analyzed. A sample size of 40 patients was predicted based on similar studies published regarding the assessment of parental satisfaction with various aspects of the healthcare system via telephone interviews.^4,5^ This was further confirmed when data saturation, the point at which telephone calls failed to generate new themes, was reached within the 40 parents interviewed.

### Data Analysis

Qualitative inductive content analysis was conducted by a single research team member (ED) to identify themes emerging from the interview responses. As findings could be shaped by the experiences and beliefs of the research member performing the analysis, data were further reviewed by the full team, and iterative revisions were made until consensus was achieved.

## Results

Of the 78 families contacted, 16 parents declined to participate while 40 parents agreed, and the remaining 22 parents could not be reached. See Table 1 for patient characteristics.

**Table 1.** Patient Characteristics.

Characteristics:	n (%)
Total patients	40
Male	26 (65%)
Female	14 (35%)
Age at the time of injury (years)	
Mean	11.65
Range	5-16
Comorbidities	
Diabetes type 1	1 (2.5%)
Seizure disorder	1 (2.5%)
Asthma	1 (2.5%)
Adenoid hypertrophy	1 (2.5%)
Injured finger	
Thumb	6 (15%)
Index finger	3 (7.5%)
Middle finger	7 (17.5%)
Ring finger	5 (12.5%)
Small finger	19 (47.5%)
Fracture location	
Metacarpal	10 (25%)
Proximal phalanx	19 (47.5%)
Middle phalanx	9 (22.5%)
Distal phalanx	2 (5%)
Fracture type	
Phalanx base (including Salter Harris)	24
Phalanx midshaft	2
Phalanx head	1
Metacarpal base	2
Metacarpal midshaft	3
Metacarpal neck	1
Metacarpal head (including Salter Harris)	2
Volar plate	4

All 40 parents (100%) reported complete satisfaction with the SFP. Additionally, all 40 parents (100%) felt that their expectations regarding treatment were met by the OTs who provided care. Although overall satisfaction was unanimous, a small amount of constructive feedback was offered. Specifically, 3 parents raised concerns about the splint material, describing it as too malleable or uncomfortable for their children. Despite this, overall, the clinic experience was described as “amazing,” “impressive,” “perfect,” “kid friendly,” and “professional.”

The thematic analysis identified 4 topics directly relating to parental satisfaction of the SFP: (1) communicating clearly, (2) creating a calm and comfortable environment, (3) setting expectations, and (4) streamlining clinical processes. See Table 2 for representative quotes of these themes.

**Table 2.** Direct Quotations of Parental Feedback.

Theme	Quote
Communicating Clearly	“The OT was really good at talking with my child and she kept me in the loop as well.”
	“The instructions were really clear.”
	“The OT explained everything to me and she was really good with my son. She explained all of the information and then demonstrated the different exercises that he needed to do.”
	“The OT did a good job of communicating with my son, she made sure that my son understood the exercises.”
	“Everything was straightforward, but any questions that we did have were perfectly answered.”
	“The OT told us how to use the cast, how we have to treat the cast, what we have to look for with respect to healing. We got exercises for my son and she actually did the exercise with him, so he understood how to do it. We went through each and every one of the exercises so that he could do them by himself.”
	“The OT answered all the questions, even if it was from the second child that I had with me or my son. She was very helpful.”
	“The OT that was helping my daughter was very informative and pretty much just helped her feel that she was going to be okay. She felt that she could come back at any time and see him and do like alterations to her customized splint.”
	“My daughter was getting a rash from the cast and I called the clinic for help and I got called back right away. I knew who to call.”
Creating a Calm and Comfortable Environment	“The OTs were really good towards my son, they made him feel comfortable and he has anxiety so that was really nice.”
	“I lucked out with the health care providers that were really good with children.”
	“All the OTs were super friendly and put my child at ease.”
	“The whole team that we dealt with was absolutely amazing.”
	“Everybody was super friendly”
	“The OTs were just so friendly and just amazing, very easy to understand and patient.”
	“They were great with my son. He was only 5 and he was a little bit scared going in and the OTs were wonderful.”
	“It was a comfortable atmosphere for my daughter. She was just 5 at the time of the visit, and it can be kind of scary I think sometimes going to the hospital, but the OT was very kind and good with children, and we were definitely satisfied with the care we received there.”
	“I think it was just great that it was all very kid-friendly, and it made my son feel very comfortable.”
	“It was so comfortable for my child, seriously.”
Setting Expectations	“She explained everything, took her time to walk us through what to expect, and how long to wear the splints.”
Streamlining Clinical Processes	“The clinic was very seamless and everything was done in a timely manner.”
	“I actually got called for the appointment, maybe even the next day or the day after we were in the ER, and we saw somebody at Children's within like a couple of days of that call.”
	“We got our appointment very quick, less than a week after the injury.”
	“I waited less than 5 minutes to be brought into the appointment, I was very amazed by that. I was expecting to be there all day.”
	“Literally no notes. I was very impressed by how efficient it was and how quick it was.”
	“I received a call with an appointment the next day.”
	“Everything ran really smooth and like a well-oiled machine.”
	“I was surprised how fast I got the call.”
	“They were on time and efficient.”
	“It was very quick, very professional.”
	“We were called less than 24 h after being in the ER”
General Feedback	“It wasn't until we went to Children's that we felt like we were being helped.”
	“It was a really good experience.”
	“After he went to Children's, I felt much better that he was receiving the care he needed. After his appointment, I felt very reassured with everything.”
	“Our care at Children's was wonderful compared to the ER.”
	“They went above and beyond.”

### Communicating Clearly

Parents consistently praised the OTs for their “vast knowledge” and ability to simplify complex medical information. They reported receiving “very clear,” “informative,” and “straightforward” instructions that facilitated understanding. In addition to verbal explanations, parents valued the OT's hands-on demonstrations, which enhanced their ability to implement exercises at home, ultimately boosting rehabilitation outcomes. The OTs were also commended for their responsiveness to questions from all family members, “taking their time to walk [parents] through what to expect,” even when facing time constraints or language barriers. Following care, 38 parents (95%) reported a clear understanding of both the diagnosis and treatment plan provided by the OTs, while 2 parents (5%) expressed confusion. Of the 2 parents who were confused, one was unclear about the splint material used and the other was confused as to why their child was not given a follow-up appointment after recovery.

### Creating a Calm and Comfortable Environment

Parents appreciated the friendly demeanor of the OTs, describing them as “kind,” “super-friendly,” “good with children,” “patient,” “helpful,” and “polite.” This approachable demeanor allowed young patients to feel more “comfortable” and “calm” in the clinical setting. Furthermore, parents felt secure in the healthcare system, confident that they “could reach out if they needed” further care, due to the kindness of the OTs.

### Setting Expectations

At discharge, families were advised that full recovery, defined as return to all normal school and extracurricular activities without a splint, would take 6 weeks. Of the 40 patients interviewed, 27 (67.5%) reported a full recovery within 6 weeks while 12 patients (30%) reported recovery taking 7 to 10 weeks, and one patient's family could not recall their exact recovery timeline. A plastic surgeon (RH) contacted each family whose recovery was greater than 6 weeks via telephone and found that 6 out of the 12 families (50%) were satisfied their child had healed within a reasonable time frame, despite being outside of the target protocol recovery timeline. Half of the 12 patients with delayed recovery also had clear reasons for the extended healing time: 3 returned to sport or other high-impact activities prior to the recommended timeline from OTs, one was incorrectly triaged (ie, a complex fracture incorrectly triaged as a simple fracture), one incurred a second, unrelated fracture to the same finger, and one presented late to hand therapy.

At the time of the telephone interview, each patient was between 2 and 7 months post visit to the OT. One of the questions in the survey asked families whether their child was still having any residual pain at the fracture site. Of the 40 patients interviewed, 36 (90%) reported no persisting pain or concerns at the site of the fracture. The 4 patients who reported pain were subsequently contacted by a plastic surgeon (RH) for more details. This revealed that all 4 children were able to do regular activities without pain; pain was only present with sports-specific movements. Further investigation revealed that all 4 patients were also highly active and had participated in competitive sports prior to the advised 6-week recovery mark.

### Streamlining Clinical Processes

Parents consistently highlighted the efficiency and punctuality of the OTs. They commended the clinic for scheduling appointments “in a timely manner” and described the clinic's operations as running “really smooth, like a well-oiled machine.” After receiving a referral, the hand fracture clinic is responsible for contacting parents to schedule an appointment within 2 weeks. Among the 40 patients, 33 parents (82.5%) reported being contacted by the clinic to arrange the appointment, while 6 parents (15%) chose to initiate the call themselves, and one parent was unsure of how the appointment was scheduled. Of the 33 parents who received a call from the clinic, 25 (75.8%) were contacted within the 2-week time frame following their referral from primary care. Only 3 parents (9%) reported waiting longer than 2 weeks for the call, and the remaining 5 parents could not recall when they received it.

## Discussion

The study aimed to evaluate parental satisfaction of the SFP; a unique triage protocol in which OTs, rather than physicians, provides management for simple pediatric hand fractures. Parents reported exceptionally high levels of satisfaction with the SFP and most patients recovered within the expected time frame, underscoring the protocol's efficacy. These findings not only validate the success of an OT-led model but also emphasize its potential to reduce healthcare system burden while maintaining high-quality, patient-centered care.

### Communicating Clearly

Effective communication is a critical component of ensuring that parents feel informed and confident in the care their child receives. In this study, parents praised the OTs for their ability to convey complex medical information in an accessible, straightforward, and child-friendly manner. Systematic reviews have shown that while surgeons tend to be thorough in delivering technical information, they often neglect the emotional and relational aspects of care, such as empathy and rapport building.^6,7^ In contrast, OTs in this study consistently demonstrated both technical clarity and interpersonal warmth, a dual strength that may help explain the high satisfaction rates reported. This highlights the importance of communication that is not only clear and informative but also compassionate and autonomy-supportive when building patient and parent trust and satisfaction.

### Creating a Calm and Comfortable Environment

Parents in this study consistently praised the demeanor of the OTs, referring to them as approachable, child friendly, and polite. As discussed, many systematic reviews have shown that surgeons fall short when building rapport with patients and parents due to their focus on the technical part of care.^6^ Literature consistently finds that positive demeanor, approachability, and the ability to develop rapport are critical components of patient satisfaction.^7^ The OTs in this setting demonstrated this characteristic, allowing parents to feel supported and children to feel comfortable through their demeanor. Their demeanor is part of what contributed to the exceptional satisfaction levels reported and may not have been as easily achieved within the traditional, physician-led model of care. These findings support the growing emphasis on interprofessional care models in pediatric rehabilitation, where providers such as OTs are not only clinically competent but uniquely skilled in delivering family-centered, emotionally supportive care.^8^ Recognizing and leveraging these strengths may be essential in designing patient care pathways that optimize both outcomes and satisfaction.

### Setting Expectations

The recovery data from this cohort highlight both the overall effectiveness of the SFP and the quality of care provided by the OT team. Most patients (67.5%) achieved full recovery within the expected 6-week timeline, aligning with the discharge guidance provided. Notably, even among those whose recovery extended beyond 6 weeks, half still felt their child had healed within a reasonable time frame, suggesting that family satisfaction with care remained high despite variations in recovery timelines.

The reported recovery timelines in this study are similar with the literature. An Australian study published in 2016 reported that average healing times for pediatric hand fractures vary by age: 6 weeks for children aged 5 to 8, 8 weeks for children aged 9 to 11, and 10 weeks for children aged above 12.^9^ The mean age in this study was 11.65 years, placing the patients closer to the 8 to 10 week expected healing window according to the Australian study. Despite this, most of our patients recovered within our allotted 6 week time frame, highlighting the effectiveness of the SFP.

Delayed recovery (30%) and residual pain (10%) were largely attributable to patients engaging in competitive sports prior to completing the advised rest period. This suggests that the SFP is highly effective when patients adhere to the recommended guidelines. The challenge of pediatric patient compliance is well-documented in prior literature. The same Australian study found that 45% of children were nonadherent to activity recommendations following hand trauma.^9^ An American study on pediatric compliance in weight bearing restrictions post lower extremity fracture was 15%.^10^ These findings collectively highlight the importance of clear communication and education around activity restrictions, as adherence plays a crucial role in optimizing recovery outcomes in pediatric populations.

### Streamlining Clinical Processes

The smooth and efficient operation of the hand therapy clinic both prior to and during the appointment emerged as a key factor contributing to parental satisfaction. In team-based models across various medical disciplines, such as cardiology, efficiency has been directly linked to improved satisfaction.^8^ A study by Sharpe et al demonstrated that implementing a structured collaborative healthcare model resulted in more timely access to care, which in turn significantly enhanced both patient satisfaction and outcomes.^8^ Similarly, in this study, the streamlined care delivery provided by the OT led clinic contributed not only to satisfaction but also to parents’ confidence in the healthcare system. By reducing wait times and minimizing logistical friction, the clinic fostered a sense of trust and reliability that was clearly appreciated by families.

### Limitations

Although this study provides valuable insights into parental satisfaction with the SFP, data were based on a self-reported interview, which are inherently subject to bias. Recall bias could affect parents’ recollection of specific details, such as the exact timing of appointments or the nature of the information received. Although every effort was made to collect accurate and honest feedback, these biases may influence the study's findings.

## Conclusion

The SFP, led by OTs, achieved unanimous parental satisfaction while supporting timely recovery and minimizing return visits. Key drivers of satisfaction included (1) communicating clearly, (2) creating a calm and comfortable environment, (3) setting expectations, and (4) streamlining clinical processes. These findings highlight the value of OT led care in managing simple pediatric hand fractures and support broader implementation of interprofessional, patient-centered care models at other institutions.

## Supplemental Material

sj-docx-1-psg-10.1177_22925503251414393 - Supplemental material for Parents Appreciate Streamlined Care From Occupational Therapists for Their Child's Simple Hand Fracture: A Qualitative Study Supplemental material, sj-docx-1-psg-10.1177_22925503251414393 for Parents Appreciate Streamlined Care From Occupational Therapists for Their Child's Simple Hand Fracture: A Qualitative Study by Emily L.M. Dalton, Thomas R. Cawthorn, Yoga Dhanapala, Frankie O.G. Fraulin, A. Robertson Harrop, Karen Hulin and Rebecca L. Hartley in Plastic Surgery

**Figure:** Video 1.
