# Supplementary material for: Parents Appreciate Streamlined Care From Occupational Therapists for Their Child's Simple Hand Fracture: A Qualitative Study
Source: Plast Surg (Oakv). 2026 Feb 5:22925503251414393. Online ahead of print. doi: 10.1177/22925503251414393 (PMC12875891; doi:10.1177/22925503251414393)
Supplement: sj-docx-1-psg-10.1177_22925503251414393 - Supplemental material for Parents Appreciate Streamlined Care From Occupational Therapists for Their Child's Simple Hand Fracture: A Qualitative Study [file sj-docx-1-psg-10.1177_22925503251414393.docx]

**Appendix 1:** Telephone Interview Script

First point of care:

1. Where did you first take your child for assessment after the injury? (e.g., emergency room, urgent care, family doctor)
2. At this first visit, what care did your child receive for their hand fracture? (e.g., splint or cast, closed reduction, buddy taping)
3. Did you see any other health care provider between this first visit and your follow up with our clinic?
4. What was your understanding about what was going to happen after this first visit?
   1. What type of healthcare provider did you think you were going to see for follow-up?
5. Did you receive any written instructions?
   1. Tell us what instructions you received
   2. Did you receive a phone number to call if you needed further information?

Getting your hand therapy appointment:

1. How was your follow-up appointment organized/booked?
2. If you received a phone call to book an appointment, when did you get that call?
3. What was your understanding about what was going to happen at your child’s next health care appointment?

Hand Therapy:

1. Did your child’s appointment with the hand therapist meet your expectations?
2. Were you confused about any aspects of your child’s care?
3. Did you expect to see a surgeon or physician at any point in follow-up?

Outcome:

1. How long did it take for your child to return to all normal activities after the injury?
   1. Was this timeline shorter or longer than expected?
   2. Does your child have any ongoing problems with their hand?
2. Did your child have to return to the emergency room or urgent care center for the same fracture at any point?
3. Did you have to see your family doctor at any point regarding the fracture?
4. Did you have to see another physician at any point?

Suggestions for improving care:

1. Was there anything that could have been done, during any phase of the care, that would have made the process better?

Thank you so much for your time and cooperation.
